# Validation, reproducibility and safety of trans dermal electrical stimulation in chronic pain patients and healthy volunteers

**DOI:** 10.1186/1471-2377-10-5

**Published:** 2010-01-13

**Authors:** Remigiusz Lecybyl, Juan Acosta, Joydeep Ghoshdastidar, Kinga Stringfellow, Magdi Hanna

**Affiliations:** 1 King's College London; Pain Clinical Research Hub, Denmark Hill Campus, London, UK; 2 Royal Free Hospital; Anesthesiology Department, Pond Street, London, UK; 3 Queen's Mary Hospital; Anesthesiology Department, Frognal Avenue, Sidecup, UK; 4 Lewisham University Hospital; Anaesthesiology Department; Lewisham High Street, London, UK

## Abstract

**Background:**

Surrogate pain models have been extensively tested in Normal Human Volunteers (NHV). There are few studies that examined pain models in chronic pain patients. Patients are likely to have altered pain mechanisms. It is of interest to test patient pain responses to selective pain stimuli under controlled laboratory conditions.

**Methods:**

The Institutional Ethic Committee approved the study. 16 patients with chronic neuropathic radiculopathy and 16 healthy volunteers were enrolled to the study after obtaining informed consent. During electrical stimulation (150 minutes for volunteers and 75 minutes for patients) the following parameters were measured every 10 minutes:

Ongoing pain: Visual Analogue Scale (VAS) and Numeric Rate Scale (NRS)

Allodynia (soft foam brush)

Hyperalgesia (von Frey monofilament 20 g)

Flare

For each endpoint, the area under the curve (AUC) was estimated from the start of stimulation to the end of stimulation by the trapezoidal rule. The individual AUC values for both periods were plotted to show the inter- and intra-subject variability. For each endpoint a mixed effect model was fitted with random effect subject and fixed effect visit. The estimate of intra-subject variance and the mean value were then used to estimate the sample size of a crossover study required to have a probability of 0.80 to detect a 25% change in the mean value. Analysis was done using GenStat 8^th ^edition.

**Results:**

Each endpoint achieved very good reproducibility for patients and NHV. Comparison between groups revealed trends towards:

Faster habituation to painful stimuli in patients

Bigger areas of hyperalgesia in patients

Similar area of allodynia and flare (no statistical significance)

**Conclusion:**

The differences demonstrated between patients and NHVs suggest that the electrical stimulation device used here may stimulate pathways that are affected in the pathological state.

## Background

Experimental pain models are key tools for improving our understanding of pain mechanisms and play an increasingly important role in providing early evidence of clinical efficacy, evaluating new analgesic compounds and establishing a link between preclinical and clinical pain research. Assessment of clinical pain presents a unique challenge compared to other major health conditions, such as heart disease or cancer, which can be detected by objective biological measurements, where the diagnosis of chronic pain depends upon subjective reports by patients on the presence and intensity of pain. However, comparable reports on sensory attributes cannot be obtained from laboratory animals without language skills [1].

The need for increased translational research in the field of pain research was highlighted recently following the development of the 'selective substance P receptor antagonist class' which was efficacious in a wide range of animal models, but failed to alleviate pain in patients [2,3]. Ultimately, only patient studies can truly validate the clinical efficacy of new analgesics, however these clinical trials are very expensive for early phase drug evaluation. Surrogate or experimental pain models in human volunteers represent a more economical approach than patient clinical trials, as they require a smaller number of subjects and are therefore less expensive. This is achieved whilst maintaining good experimental control of the model itself. Nonetheless, surrogate pain models have suffered from a lack of reproducible data and inconsistency of pharmacological characterization.

The ultimate goal of modern pain assessment is to obtain a better understanding of mechanisms involved in pain transduction, transmission and perception under normal and pathophysiological conditions [4]. Surrogate human pain models should use standardized methodology to produce reproducible endpoints. Methodology should be appropriate and ethical for both patients and volunteers.

There is a long and well-documented history of electrically induced pain, sensitization and flare [5-9]. Nevertheless, there are a number of barriers that limit the wider application of electrical pain models in pain research. Earlier methods utilized an "almost unbearable intensity" of current [6,9]. The introduction of intra-dermal stimulation led to a reduction in the intensity of pain needed to evoke sensitization but required invasive procedures. This included drilling an epidermal hole [5], inserting custom made intradermal needles [8] or inserting sterile custom made intradermal electrodes [7]. Intradermal electric pain models [8] are able to produce ongoing pain, hyperalgesia and flare in reproducible patterns. Intradermal electrical pain model was characterized by many pharmacological agents including: intravenous alfentanil, S(+)-ketamine, lidocaine [8], tramadol and acetaminophen [10], Pregabalin and Aprepitant [11]. Additionally this model was extensively used to investigate mechanisms of hyperalgesia [12-17]. The use of intradermal electrodes that need to be inserted under ice analgesia could limit the wider use of this model. The invasive nature of the procedure is likely to exclude utilizing this model in chronic pain patients on practical and ethical grounds. The Transdermal Electric Stimulation (TDES), where it is free from skin insertion, is the next step in the development of electric pain models. The new model would offer a safer testing of patients as well as healthy volunteers.

Neuropathic patients are already suffered from altered pathways and central sensitization. Our hypothesis is that the sympathology may be localized in some area or another. Global central sensitization is likely to produce abnormalities anyway within central nervous system which had never been explored before

The aims of this study were 1) to evaluate reproducibility, safety and tolerability of pain and sensitization produced by TDES in (a) patients with chronic lumbo-sacral pain with radicular neuropathic features and (b) Normal Healthy Volunteers (NHV) and 2) compare the differences in the pain endpoints of hyperalgesia and allodynia between those two groups.

## Methods

### Study protocol

Two experiments one on patients and the other on NHV with similar experimental paradigm was performed. Each of them consisted of two identical sessions, at least one week apart, and was conducted to assess reproducibility and tolerability of TDES. 16 healthy volunteers were recruited for the first experiment and 16 patients suffering from lumbosacral pain with radicular neuropathic features were recruited for the second experiment. Subjects were seated comfortably in a reclining chair, with the forearms resting with the volar side up. Room temperature was controlled at 20 - 24 degree Celsius. The same investigator performed both sessions for each subject. The assessments for the NHVs study were performed by a single investigator (investigator 1); whereas the assessments for the patients study were performed by two different investigators: investigator 1 (same as for the NHVs study) assessed patients ID01 to ID06, ID13 and ID14, and investigator 2 assessed patients ID7 to ID12.

### Volunteers

After approval from the Institutional Ethic Committee 16 male NHVs (mean age: 27.8 years, range 22-38) were recruited and 16 male patients (mean age: 42.6 years, range: 39-66) diagnosed with chronic lumbosacral pain with radicular neuropathic features were recruited. If English was not the subjects' first language, the investigator would assess their understanding of the process, and make a decision on whether they could give informed consent before entering them into the study. Informed consent was obtained from each volunteer.

Chronic pain was defined as pain with intensity of at least 5 on the 0-10 Numeric rate Scale (NRS) scale with duration of at least 3 months. Diagnosis was based on expert opinion and neuropathic features were additionally confirmed by DN4 questionnaire (score equal or higher than 4 out of 10) [18,19]. Because the study aimed at assessing reproducibility, only patients on stable medications were included. Subjects were advised not to have any coffeine or alcohol in the evening prior to each visit. NHVs where also asked not to take any 'over the counter' analgesic, and patients not to take any as required analgesic, for 24 hours prior to each study visit. Due to the nature of the transdermal device, any subjects with skin problems, such as eczema or psoriasis, were excluded. Any subjects with a history of psychosis or psychological disease which might affect neural pathways, or hinder the patients pain perception or ability to complete the task set out in this study, or requiring psychoactive drug treatment were also excluded.

### Trans dermal stimulator

**Figure 1:**
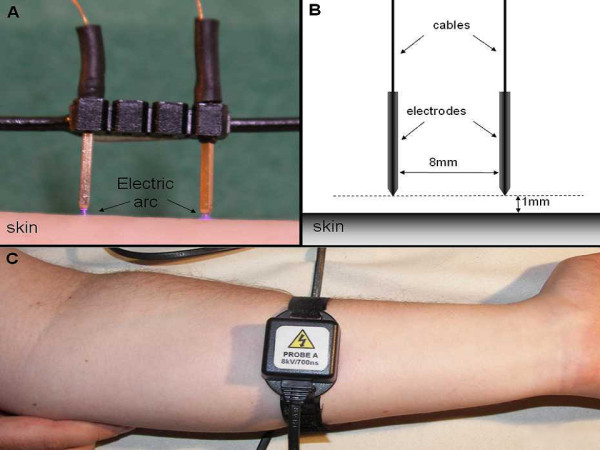
**Transdermal Electric Pain Stimulator (HV-1)**. A) Stimulator without enclosure. Electrostatic-like discharges are visible between skin and the electrodes, B) Scheme of the stimulation head, C) Stimulator is simply attached to the forearm by Velcro strap.

A recently described High Voltage Pain Stimulator (HV-1) was used [20]. HV-1 produces trains of nanosecond-range pulses with a train frequency of 2 Hz. Each train consists of a variable number of single pulses. The intensity of stimulation is defined as a number of pulses in the train. The length of train varied between 0-6 milliseconds. Constant current or Pulse Width Modulation (PWM) is impractical for nanosecond pulses in kilovolt-range due to the limitation of available electronic components. Electric pulses (similar to electrostatic discharges) are transferred between the two electrodes and skin by electrostatic discharges (Figure 1A). Single electrostatic discharge has a biphasic dumped oscillating characteristic with amplitude of 8000 Volts. The distance between the electrodes is equal to 8 mm and there is 1 mm between each electrode and the skin (Figure 1B). There is no physical contact between the skin and the electrodes. The stimulation head is attached to the forearm by Velcro straps (Figure 1C).

### Paradigm of electric stimulation

Electric stimulation was applied to the non-dominant forearm via the High Voltage Pain Stimulator (HV-1). At the beginning of each session the intensity of stimulation (number of pulses in the train) was gradually increased every 5 minutes over 15 minutes, targeting a pain rating of five on the 11-point numeric rating scale (NRS, 0 = No Pain, 10 = Maximum Imaginable Pain). This was done in order to take into account individual sensitivity to electrically evoked pain as this varies substantially across subjects. For the rest of the session (155 minutes for healthy volunteers and 75 minutes for patients) the electric intensity was kept constant. Throughout the stimulation the endpoints were measured every 5 minutes in the first 15 minutes and then every 10 minutes once the electricity intensity was constant. The paradigm of electric stimulation and measurements is graphically presented at Table 1.

**Table 1:** Experimental protocol.

Time relative to start of stimulation	0	5	10	15	25	35	45	55	65	75	85	95	105	115	125	135	145	155	165	175	185
Electrical stimulation	↔																				
Stimulus titration	x	x	x	x																	
Measurements:																					
Pain score	x	x	x	x	x	x	x	x	x	x	x	x	x	x	x	x	x	x	x	x	x
Punctatate hyperalgesia	x	x	x	x	x	x	x	x	x	x	x	x	x	x	x	x	x	x	x	x	x
Brush allodynia	x	x	x	x	x	x	x	x	x	x	x	x	x	x	x	x	x	x	x	x	x

This table shown time and events in both identical sessions

### Endpoints

Four endpoints were measured:

1. Ongoing pain. Throughout the duration of experiment subjects were asked to rate the pain sensation induced by electric stimulation using Numeric Rate Scale (NRS 0-10, 0 = No Pain, 10 = Maximal Imaginable Pain) and Visual Analogue Scale (VAS 0-100, 0 = No Pain, 10 = Maximal Imaginable Pain). Due to subjective character of pain intensity measurements two different, independent pain scales was used for better validation.

2. Flare field. The size of the visual flare field was measured as a diameter of skin redness. The skin was observed, and diameter was measured in line parallel to the axis of the forearm. The diameter was expressed in centimeters.

3. Allodynic field. The size of the allodynic field was measured as a diameter of brush evoked dynamic allodynia to a response to foam brush stimulation. The skin was gently brushed in two linear paths parallel to the axis of the forearm from distant starting points towards the stimulation site until the volunteer reported an increase in sensation related to brushing. Volunteers and patients reports this sensation as unpleasant (especially in proximity of stimulator. The diameter was defined as the distance between the most proximal and the most distal point at which volunteers' reported an increase in sensation, and was expressed in centimeters.

4. Hyperalgesic field. The size of the pin-prick punctuate hyperalgesic field was measured as a diameter of pin-prick punctuate hyperalgesia (Frey monofilament: No 15, Force = 170 mN, Diameter = 0.61 mm, Pressure = 57.8 g/mm^2^, SenseLab - Aesthesiometer, Somedic, Sweden and custom made weighted needle: Force = 200 mN, Diameter = 0.6 mm, Pressure = 70.78 g/mm^2^). The applied force was higher than the published mean mechanical pain threshold (129 mN) on the hand in NHVs [21]. There are no data available for pain threshold on forearm and hand pain treshold was used as closest approximation available. The skin was stimulated by a monofilament in two linear paths (proximal and distal) parallel to the axis of the forearm from distant starting points towards the stimulation site until the volunteer reported an increase in sensation related to monofilament stimulation. Volunteers and patients reports this sensation as unpleasant (especially in proximity of stimulator). Most subjects reports this sensation as painful. The diameter was defined as the distance between the most proximal and the most distal point at which volunteers report an increase of sensation and was expressed in centimeters.

The fields of sensitization were estimated only in one axis as preliminary data (not published) showed for some subjects sensitization on dorsal area of forearm making measurements on axis perpendicular to forearm very difficult.

### Statistical methods

For each endpoint, pain scores, visual flare, allodynia and hyperalgesia diameters, the average value over the 15 to 75 minutes-electrical stimulation period (area under the curve divided by time, AUC/t) was calculated and used as a summary measure; a mixed effect model with subject as random effect, and visit as fixed effect was fitted to each endpoint summary measure. Model assumptions were checked by plotting residual plots. The back-transformed ANOVA-adjusted means values for each group of subjects were derived together with the 95% confidence intervals based on the standard error of the mean. From each model, the estimates of intra-subject and inter-subject variances were obtained to assess endpoint reproducibility. Analysis was completed using GenStat 8th edition http://www.vsn-intl.com.

## Results

### Subjects

All data from the 16 male NHVs and the 16 patients were included in the analyses. Data for the second visit was missing for one NHV due to this subject moving to another city and for two patients, one due to repetitive non-compliancy with appointment times and the other due to technical error for the other.

### Tolerability

The stimulation was well tolerated by all subjects. Despite no physical contact between the electrodes and skin, some injury was created by the electric discharge but for most volunteers the injury was almost undetectable. One volunteer and two patients has obvious injury bigger than 1 mm and less than 2 mm. All injuries healed spontaneously in few days. Future studies could include skin microphotography for formal injury assessment.

### Electrical current intensity

**Figure 2:**
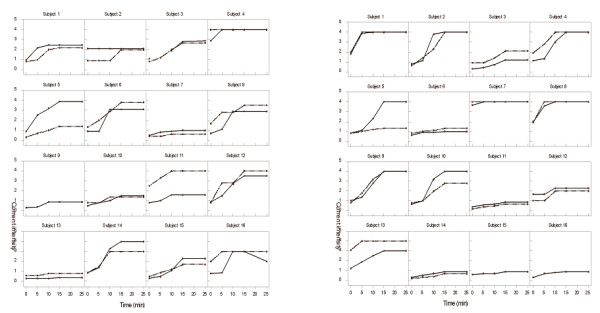
**Individual time courses of electrical current intensity during the ramping-up period (0-15 min) by visit**. Thick lines, Visit1; dotted line, Visit2; a. Healthy volunteers; b. Patients.

During the ramping-up period, the current intensity was increased until reaching a pain score of 5 on the NRS scale or until reaching the maximum current allowed, a current intensity of 4 (Figure 2). Overall, patients appeared to require a higher current intensity to reach a pain score of 5 on the NRS scale; six out of the 16 patients (Subjects 1, 2, 4, 7, 8 and 9) reached the maximum current intensity on both visits for the continuous electrical stimulation as compared to one NHV (Subject 4). Figure 2 also illustrates the visit-to-visit reproducibility of the current required to achieve a pain score of 5 on the NRS scale during the titration period. Current values were very similar for both visits to the exception of one subject in each group (healthy subject 11 and chronic pain subject 5). Healthy subject 16 had to have the current lowered from 25 min onwards on their first visit because their pain reached a score of 6.

### Pain scores

**Figure 3:**
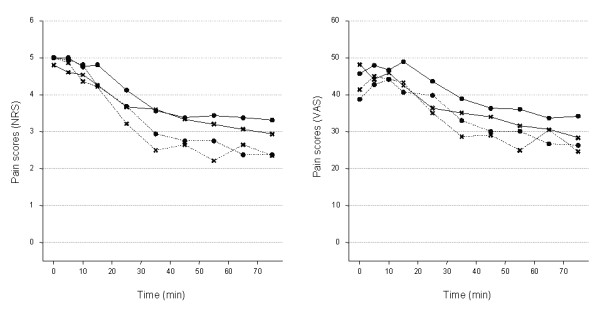
**Average time course (0-75 min) of NRS (left panel) and VAS (right panel) pain scores at each visit**. The electrical stimulation was maintained for 155 minutes in HVs; and for 85 minutes in patients. Thick line, healthy volunteers; dotted lines, patients. Circles, Visit1; Crosses, Visit2.

During the constant stimulation period, the intensity of pain gradually decreased, despite the fact that the current intensity remained constant, and after 75 minutes of electrical stimulation the pain scores were on average VAS ≥ 30 for NHVs and VAS ≥ 25 units for patients, and NRS>3 units for NHVs and NRS~2.5 units for patients. (see raw means plot on Figure 3). On stopping the electrical stimulation, the pain disappeared instantaneously (data not shown). The time course of the pain intensity appeared to be slightly different between the two groups: the painful sensation seemed to decrease at a faster rate in patients than in NHVs, and the average pain over the 15-75 min period was lower in patients (VAS, mean:30.6; adjusted s.e.:2.18 - NRS, mean:2.9; s.e.:0.12) compared to the NHVs (VAS, mean:36.4; s.e.:2.72 - NRS, mean:3.6; s.e.:0.19) (detailed in Table 2). However, the 95% CI based on the standard error of the means did overlap (Figure 3).

**Table 2:** Summary table of mixed model ANOVA estimates.

HEALTHY VOLUNTEERS							
**ENDPOINTS**	**Inter-subject SD**	**Intra-subject SD**	**ICC**	**Mean**	**Mean s.e**.	**95%CI lower**	**95%CI upper**

Pain score (NRS)	1.0	0.8	0.5	3.6	0.19	3.17	3.93
Pain score (VAS)	12.5	10.9	0.2	36.4	2.72	31.07	41.73
Hyperalgesia diameter/195 mN Von Frey hair (cm)	5.6	1.9	0.9	13.9	0.47	13.02	14.84
Allodynia diameter/coton bud (cm)	5.8	2.9	0.7	11.0	0.74	9.59	12.47
Flare diameter (cm)	2.9	2.0	0.5	6.4	0.50	5.43	7.37

**PATIENTS**							

ENDPOINTS	Inter-subject SD	Intra-subject SD	ICC	Mean	Mean s.e.	95%CI lower	95%CI upper

Pain score (NRS)	1.0	0.5	0.8	2.9	0.12	2.63	3.09

Pain score (VAS)	14.6	8.7	0.6	30.6	2.18	26.33	34.87

Hyperalgesia diameter/195 mN Von Frey hair (cm)	5.9	2.0	0.9	9.9	0.49	8.91	10.85

Allodynia diameter/coton bud (cm)	6.6	3.0	0.8	10.1	0.74	8.66	11.58

Flare diameter (cm)	2.9	2.1	0.5	6.5	0.53	5.41	7.49

### Visual flare diamete

**Figure 4:**
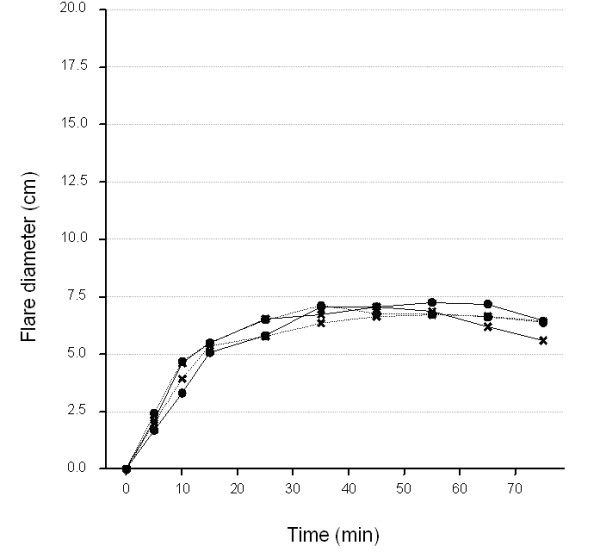
**Average time course (0-75 min) of visual flare diameter at each visit**. Thick line, healthy volunteers; dotted lines, patients. Circles, Visit1; Crosses, Visit2.

We observed an area of flare visually (redness) in all volunteers upon electrical stimulation. The diameter of flare gradually increased, reaching a maximum at 50 minutes, and then gradually decreased independently of the electrical stimulation to disappear by 100 minutes post-stimulation (Figure 4 and data not shown). The visit-to-visit reproducibility of the visual flare diameter for each subject, as assessed by plotting individual time courses, was good (data not shown). No difference was observed in the mean visual flare over the 15-75 min period or in the flare variability between NHVs (mean:6.4; s.e.:0.50) and patients (mean:6.5; s.e.:0.53) after adjustment for subject and visit effect (Table 2).

### Allodynia/Secondary hyperalgesia

In the present study, one NHV did not experience hyperalgesia and two patients experienced hyperalgesia only to a limited extend. Interestingly, one of these patients did experience a strong allodynic response, whereas the other one did not experience any. Overall the diameter of allodynia was within that of secondary hyperalgesia.

**Figure 5:**
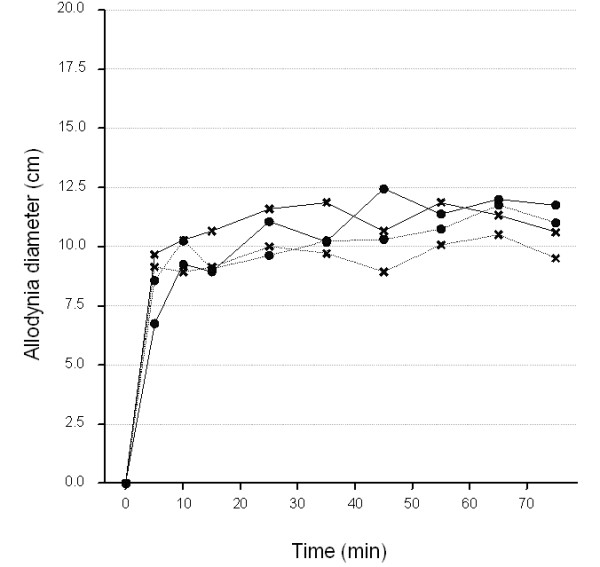
**Average time course (0-75 min) of allodynia diameter at each visit**. Thick line, healthy volunteers; dotted lines, patients. Circles, Visit1; Crosses, Visit2.

**Figure 6:**
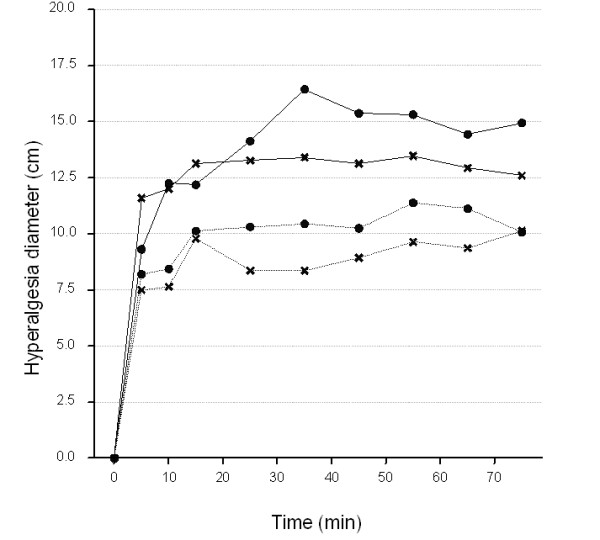
**Average time course (0-75 min) of hyperalgesia diameter at each visit**. Thick line, healthy volunteers; dotted lines, patients. Circles, Visit1; Crosses, Visit2.

Diameter static pin-prick hyperalgesia and dynamic brush allodynia followed a similar time course, gradually increasing and reaching their maxima in 35 minutes, achieving an almost plateau effect (Figures 5 &6), then gradually decaying during the post-stimulation period (stimulator switched off, data not shown). The time courses of these endpoints appeared to be similar for the two groups of subjects studied.

From the raw mean plots, the ANOVA-adjusted mean plots and the ANOVA model means and variance components estimates, there did not seem to be any evidence that the mean allodynic response over the 15-75 min period, and the inter- and intra-subject variabilities differed noticeably between NHVs (mean:11.0; s.e.:0.74) and patients (mean: 10.1; s.e.:0.74) (Figure 5 and Table 2).

In contrast, applying the same analysis to the hyperalgesia data (Figure 6) suggested that the mean hyperalgesic response over the 15-75 min period was lower in patients (mean:9.9; s.e.:0.49) when compared to NHVs (mean:13.9; s.e.:0.47). The inter-subject and intra-subject variabilities were quite similar for both study groups (Table 2).

## Discussion

Over the last decade, it has been recognized that the traditional way of drug development (three phases) can not be sustained and a new two phase has been suggested. An exploratory phase where a proof of concept is required followed by a full development program. A validated model that can act as a surrogate marker, would be a useful step in making the exploratory program both efficient and meaningful [22]. The use of surrogate markers to distinguish between pain mechanisms may facilitate the conduct of clinical trials and the development of new treatment strategies. Study groups or subgroups that address pain mechanisms may be distinguished ad hoc by using surrogate endpoints, and these may be used to evaluate patients' responses to treatment. Model to act as a surrogate marker, should produce endpoints which are associated with and pathophysiologically related to clinical outcome. A pain model should be accurately validated through rigorous reproducibility for each end point for it to be a potential Biomarker.

Preclinical animal models have provided many crucial insights in our understanding of pain mechanisms. Nonetheless, their predictive value has increasingly come to be questioned. In addition, relying totally on NHVs models rather than patients' models may lead to similar questionable predictability as prompted by animal models.

These current studies have demonstrated that Transdermal Electric Stimulation (TDES) can produce, robust and highly reproducible data for ongoing pain, hyperalgesia and allodynia in NHVs and patients suffering chronic lumbo-sacral pain with radicular neuropathic features. Transdermal Electric Stimulation (TDES) was safe and well tolerated in both groups.

TDES has produced the features of central sensitization that are associated with chronic neuropathic pain syndrome. The spread of the pain and hypersensitivity from the site of injury, the amplification if the pain beyond the peripheral nerve distribution and the brush evoked allodynia are all common manifestation of chronic pain and now recognized as a result of central sensitization [23].

*Hyperalgesia *was the most reproducible endpoint in both NHV and patients in this study. Comparing the data of hyperalgesia induced in patients and NHV, our results seem to imply there is a significant difference between patients and NHVs in the size of hyperalgesia field, being smaller in patients. This interesting observation may be explained either by the fact that the nociceptive pathways in chronic pain patients have been modified, so the response to a further stimulation is reduced or by the chronic use of medications by patients can alter the extent of further sensitization. Group of patient was heterogeneous, not age-matched to volunteers which could have confounding the results.

*Allodynia *was marginally less reproducible than hyperalgesia. It was postulated that the mechanisms of allodynia are distinct from the mechanisms of hyperalgesia[24]. In this model we observed a different time trend for allodynia and hyperalgesia. Unlike for hyperalgesia, there did not seem to be any difference in allodynia field between patients and NHVs.

*Ongoing pain *was observed in both patients and volunteers with some fading. Several theories have been put forward for this phenomenon: depletion of neurotransmitters, habituation of nerve fibres, long-term depression [25] and activation of descending inhibitory pathways [13]. It is interesting to note that dynamics of fade were different in NHVs and patients; this could imply that chronic pain-modifying mechanisms are involved in pain fading.

After the stimulator was switched off, pain disappeared immediately. In contrast, the field of sensitization (hyperalgesia and allodynia) was observed to decay gradually over a 30 minute period of post-stimulation. Interestingly, the decay of sensitization after switching off the stimulator was much slower in the patient group, which further indicates the involvement of the central sensitization processes (data was not shown).

*Visual flare *was very similar for both groups of subjects. The production of a visible flare is believed to be strictly due to peripheral mechanisms; therefore it was not anticipated that a difference would be observed between NHVs and patients with neuropathic pain who are believed to suffer from an alteration occurring at the CNS level.

Hyperalgesia and allodynia represent different features of central sensitisation. It appears that in this model particularly for patients rather than volunteers that the mechanism involved may be totally separate. As the result of that hyperalgesia differences appears between patients and between volunteers where allodynia as the separate pathway and a possible separate pathophysiological mechanism appeared to be different. This is an interesting finding from particularly this study, which shows the importance of analysing hyperalgesia and allodynia as separate and highlight potential differences between patient and volunteers.

The multi modal nature of the response in this model may make it ideal for use in the early phases of drug development. Differences in time trends for pain, hyperalgesia, allodynia and flare suggests that this pain model would allow us to separately observe the mechanism involved in the above processes. The study design was to assess validity and reproducibility of TDES methodology. Sample size data need to be taken with caution for future drug evaluation studies for both: healthy volunteers and patients. Some pharmacological testing could require assessment of reproducibility on repeated administration of the same stimulus intensity. Table 2 included all data required by statistician to calculate future study power for paradigm using the same subjective target pain intensity in the 2nd session.

Pain is not a reflex; it is a perceptual experience with powerful emotional and motivational components. Like all sensory systems, attributes of pain such as intensity, quality, duration, location, and extension depend upon cerebral processing. This applies to both laboratory animals and humans. Chronic pain necessarily results from abnormal activity in pain transmission systems, perturbing a hyperactive/hypersensitive pain pathway. Experimental stimulation should have the potential to reveal some differences that could improve our understanding and facilitate new modalities for treatment [22].

## Conclusion

Pain is one of the therapeutic areas of greatest clinical need where there are few existing therapies and have the least well-validated models or surrogate markers. This is a first study which have tested the reproducibility and validity of transdermal electric stimulation pain model in both normal healthy volunteers and patients with neuropathic manifestation. This study has showed a very good reproducible data both in volunteers and patients with some subtle differences. The next option for utilizing this model will be use it for pharmacological drug development both for neuropathic and non-neuropathic pain medications.

## Abbreviations

AUC: Area Under Curve; NHV: Normal Healthy Volunteers; NRS: Numeric Rate Scale; TDES: Transdermal electric Stimulation; PWM: Pulse Width Modulation; VAS: Visual Analogue Scale

## Competing interests

The authors declare that they have no competing interests.

## Authors' contributions

RL: contributed equally to: study design, carry out experiments, data analysis and drafting manuscript. He read and approved the final manuscript. JA: contributed equally to: study design, carry out experiments, data analysis and drafting manuscript. He read and approved the final manuscript. JG: contributed equally to: study design, carry out experiments, data analysis and drafting manuscript. He read and approved the final manuscript. KS: contributed equally to: study design, carry out experiments, data analysis and drafting manuscript. He read and approved the final manuscript. MH: contributed equally to: study design, carry out experiments, data analysis and drafting manuscript. He read and approved the final manuscript.

## Pre-publication history

The pre-publication history for this paper can be accessed here:

http://www.biomedcentral.com/1471-2377/10/5/prepub
